# Inflammatory Response and Secondary White Matter Damage to the Corpus Callosum after Focal Striatal Stroke in Rats

**DOI:** 10.3390/ijms23063179

**Published:** 2022-03-16

**Authors:** Rafael Rodrigues Lima, Ana Carolina Alves Oliveira, Rafael Monteiro Fernandes, Priscila Cunha Nascimento, Marco Aurelio M. Freire, Walace Gomes-Leal

**Affiliations:** 1 Laboratory of Functional and Structural Biology, Institute of Biological Sciences, Federal University of Pará (UFPA), Belém 66075-110, PA, Brazil; anacarolina@ufpa.br (A.C.A.O.); faelfernandes@gmail.com (R.M.F.); priscilacunha.n28@gmail.com (P.C.N.); 2 Graduate Program in Health and Society, University of the State of Rio Grande do Norte (UERN), Mossoró 59610-210, RN, Brazil; freire.m@gmail.com; 3 Laboratory of Experimental Neuroprotection and Neuroregeneration, Unity of Morphophysiology, Federal, University of Western Pará (UFOPA), Santarém 68040-470, PA, Brazil; wgomesleal@gmail.com

**Keywords:** striatum, stroke, microglial activation, astrocytosis, myelin degeneration, oligondrocyte death, corpus callosum

## Abstract

Stroke is one of the leading causes of death and long-term disabilities worldwide, resulting in a debilitating condition occasioned by disturbances in the cerebral vasculature. Primary damage due to metabolic collapse is a quick outcome following stroke, but a multitude of secondary events, including excitotoxicity, inflammatory response, and oxidative stress cause further cell death and functional impairment. In the present work, we investigated whether a primary ischemic damage into the dorsal striatum may cause secondary damage in the circumjacent corpus callosum (CC). Animals were injected with endothelin-1 and perfused at 3, 7, 14, and 30 post-lesion days (PLD). Sections were stained with Cresyl violet for basic histopathology and immunolabeled by antibodies against astrocytes (anti-GFAP), macrophages/microglia (anti-IBA1/anti MHC-II), oligodendrocytes (anti-TAU) and myelin (anti-MBP), and Anti-Nogo. There were conspicuous microgliosis and astrocytosis in the CC, followed by later oligodendrocyte death and myelin impairment. Our results suggest that secondary white matter damage in the CC follows a primary focal striatal ischemia in adult rats.

## 1. Introduction

Stroke is one of the leading causes of death and long-term disabilities worldwide, resulting in a highly debilitating condition elicited by disturbances in the cerebral vasculature [1,2]. The main risk factors associated with stroke are high blood pressure, age, obesity, diabetes, hypertension, and smoking as well as a sedentary lifestyle [3,4,5].

Ischemic stroke accounts for approximately 85% of all described cases, with hemorrhagic stroke corresponding to the remaining 15% [1,2,3]. Hemorrhagic stroke, which is mainly responsible for death cases, arises from the rupture of a blood vessel, causing bleeding directly into the brain parenchyma [6]. The ischemic stroke, in turn, results from the obstruction of a blood vessel by a clot. It leads to the interruption of blood flow to adjacent areas of the nervous tissue, with consequent impairment of the oxygen supply and nutrients to these regions [6,7].

The pathophysiology of stroke is complex and has not yet been fully understood at all. It has been shown that excitotoxicity, inflammatory response, oxidative stress, ionic imbalance, and activation of calpains and proteases result in distinct types of cell death, including necrosis and apoptosis [8,9,10,11]. Overall, the ischemic cascade is driven by a myriad of physiological and molecular events mainly associated with inflammation and oxidative stress. These contribute to the secondary damage following the primary ischemic insult [12,13,14].

In the ischemic core, neurons and glia quickly die due to metabolic collapse following vessel rupture or obstruction [7,15]. Nevertheless, during secondary degeneration, the primary ischemic core may expand in more than 60% invading the ischemic penumbra [7,15]. A pivotal issue in experimental neuropathology is to avoid secondary degeneration of the white matter, which is the main cause of functional deficits following stroke/excitotoxic injury [16,17,18,19] and spinal cord injury (SCI) [20,21,22].

In previous studies, we have shown the microinjections of N-Methyl-D-Aspartate (NMDA) into the ventral horn of rat spinal cord caused secondary white matter damage (WM) characterized by axonal degeneration, oligodendrocyte cell death, myelin impairment and intense inflammatory response [20,21]. We also reported that microinjections of endothelin-1 (ET-1) into the rat spinal cord also cause secondary degeneration of white matter tracts [23]. This phenomenon has also been observed by our group following microinjections of NMDA into the rat striatum [16,24,25]. As the corpus callosum (CC) is an important white matter commissure connecting brain hemispheres with important functional roles, we wondered whether the CC is secondarily affected following primary damage into the dorsal striatum [26].

In the present study, we performed microinjections of ET-1 into the dorsal striatum of adult rats to study the patterns of secondary degeneration in the CC. We observed conspicuous secondary CC damage characterized by intense microglial activation, astrocytosis, oligodendrocyte cell death, and progressive myelin impairment up to 30 days after the primary striatal ischemic damage.

## 2. Results

### 2.1. Microinjections of ET-1 Induced Focal Ischemia Restricted to the Striatum

**Figure 1:**
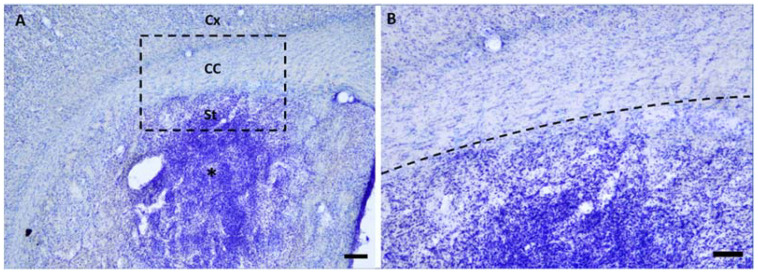
Primary focal striatal damage did not affect corpus callosum (CC) in early survival times: (**A**) Intense inflammatory response in the striatum (St, *) at 3 days following endothelin-1 (ET-1) microinjection. Primary damage is restricted to the dorsal striatum not comprising CC and cortex (Cx) (**B**) Higher magnification of image depicted in A. Scale bar: A 200 μm; B 100 μm.

**Figure 2:**
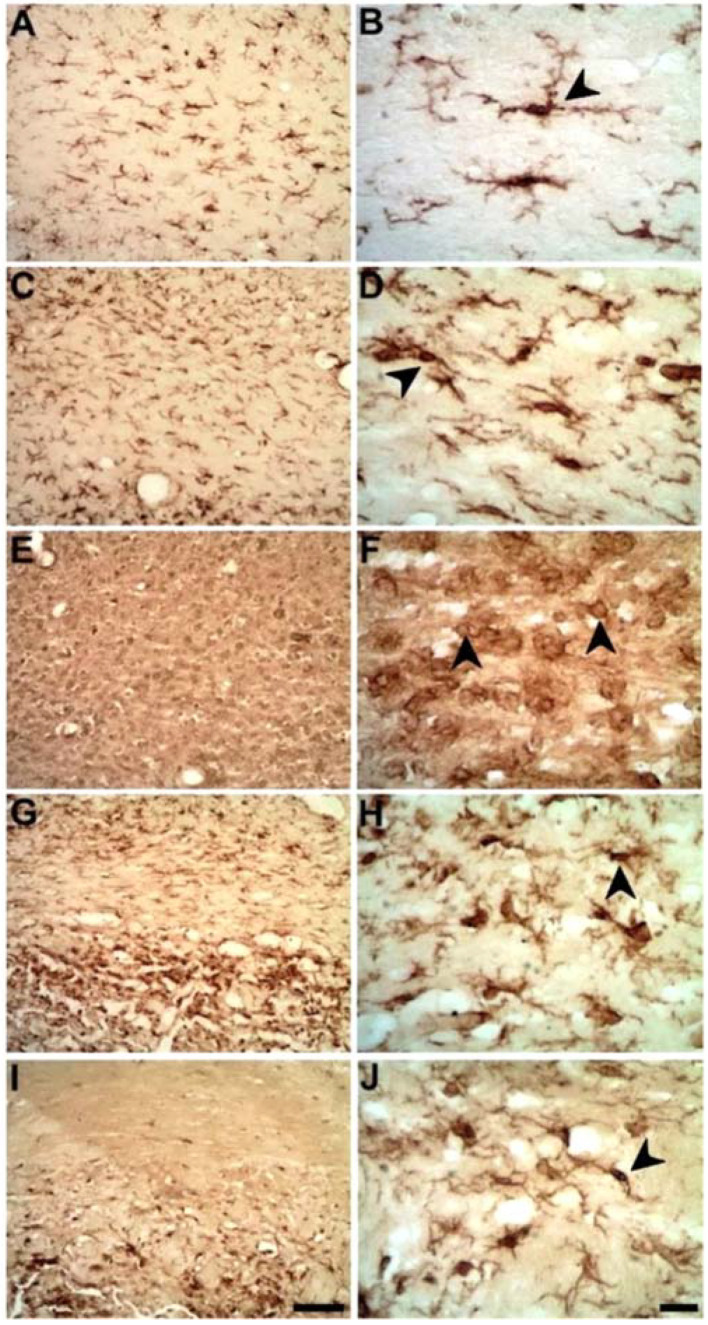
Progressive microglia activation (arrowheads) after striatal ischemia revealed by anti-Iba1 immunolabeling in the corpus callosum. Control animal injected with sterile saline (**A**,**B**) or ischemic animals injected with ET-1 at 3 (**C**,**D**); 7 (**E**,**F**); 14 (**G**,**H**); and 30 PLD (**I**,**J**). Left-sided photomicrographs in lower magnification with a scale bar of 20 μm, and right-sided ones, with higher magnification with a scale bar of 100 μm.

Microinjections of ET-1 induced focal striatal ischemia initially not affecting the CC (Figure 1). In later survival times, secondary callosal damage was observed (Figure 2), as revealed by the intense microgliosis in the callosal white matter tracts (Figure 2).

### 2.2. Microinjections of ET-1 Induced Microglial/Macrophage Activation in the Corpus Callosum

Following primary striatal damage, CC degeneration was recognized by the presence of intense microglial activation from PLD3 (Figure 2) revealed by anti-Iba1immunohistochemistry. In control animals, ramified microglia were present in the CC (Figure 2A,B). In the ischemic animals, conversely, there was progressive microglial activation (microgliosis) between 3 and 14 days after ischemic induction (Figure 2C–H), peaking at PLD7 (Figure 2C,D).

**Figure 3:**
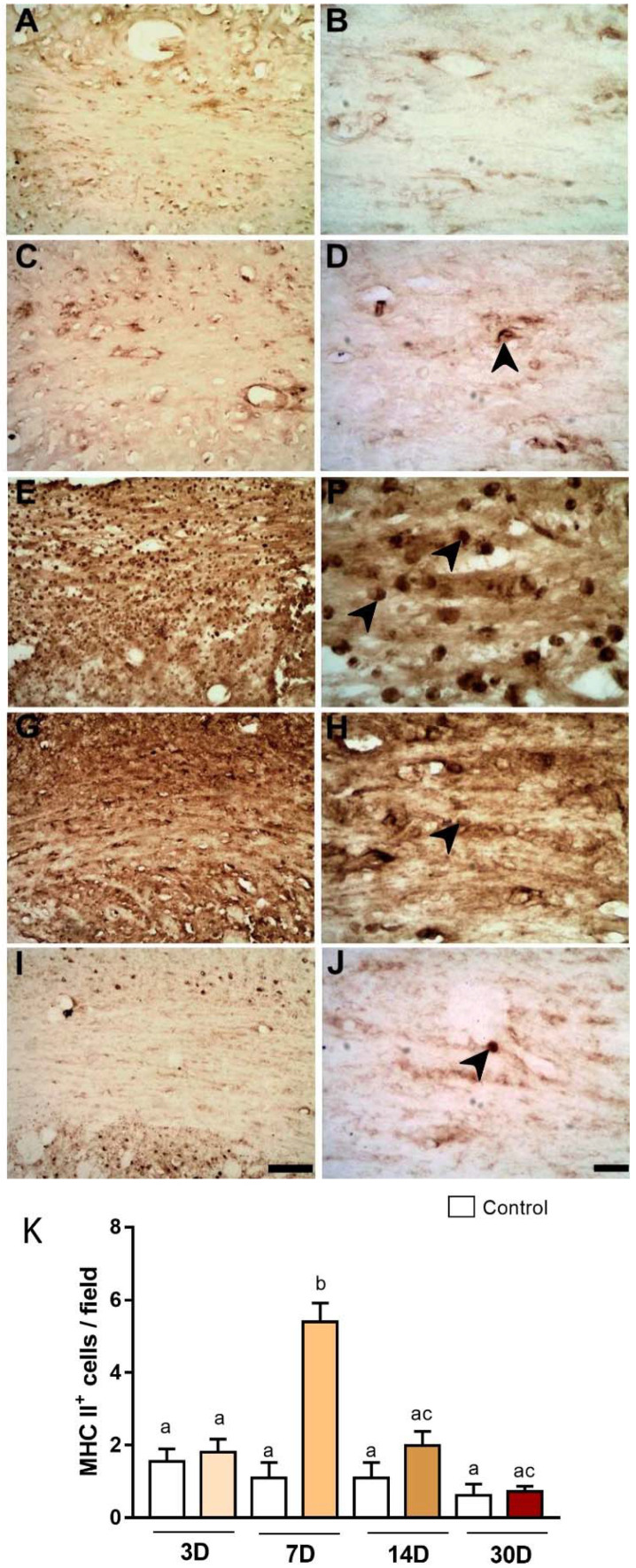
Increased immunoreactivity for MHC-II in the CC after focal striatal ischemia. Photomicrographs represent control animal injected with sterile saline (**A**,**B**) or ischemic animals injected with ET-1 at 3 (**C**,**D**); 7 (**E**,**F**); 14 (**G**,**H**); and 30 (**I**,**J**) PLDs. Maximum MHC-II immunoreactivity was present at 7 days (**E**,**F**), which has been confirmed by quantitative analysis (**K**). The results of quantitative analysis (**K**) are expressed as mean ± standard error of the mean (SEM). One-way ANOVA with Tukey’s post-hoc test (*p* < 0.05). Similar overwritten letters did not show significant statistical differences. Left-sided photomicrographs in lower magnification with a scale bar of 20 μm, and right-sided ones, with higher magnification with a scale bar of 100 μm.

Immunoreactivity for MHC class II was also investigated in all survival times: A,B, control; C,D, PLD3; E,F, PLD7; G,H, PLD14; and I,J, PLD30 (Figure 3). The maximum expression of this marker in microglia was found at PLD7 (5.40 ± 0.51 per field, Figure 3E,F), with a statistically significant difference when compared to other survival times (Control: 1.55 ± 0.34 per field; 3PLD: 1.80 ± 0.36 per field; 14PLD: 1.99 ± 0.38 per field; 30PLD: 0.73 ± 0.13 per field, *p* < 0.05, Figure 3K).

### 2.3. Degeneration of Oligodendrocytes in the Corpus Callosum after Primary Striatal Ischemia

**Figure 4:**
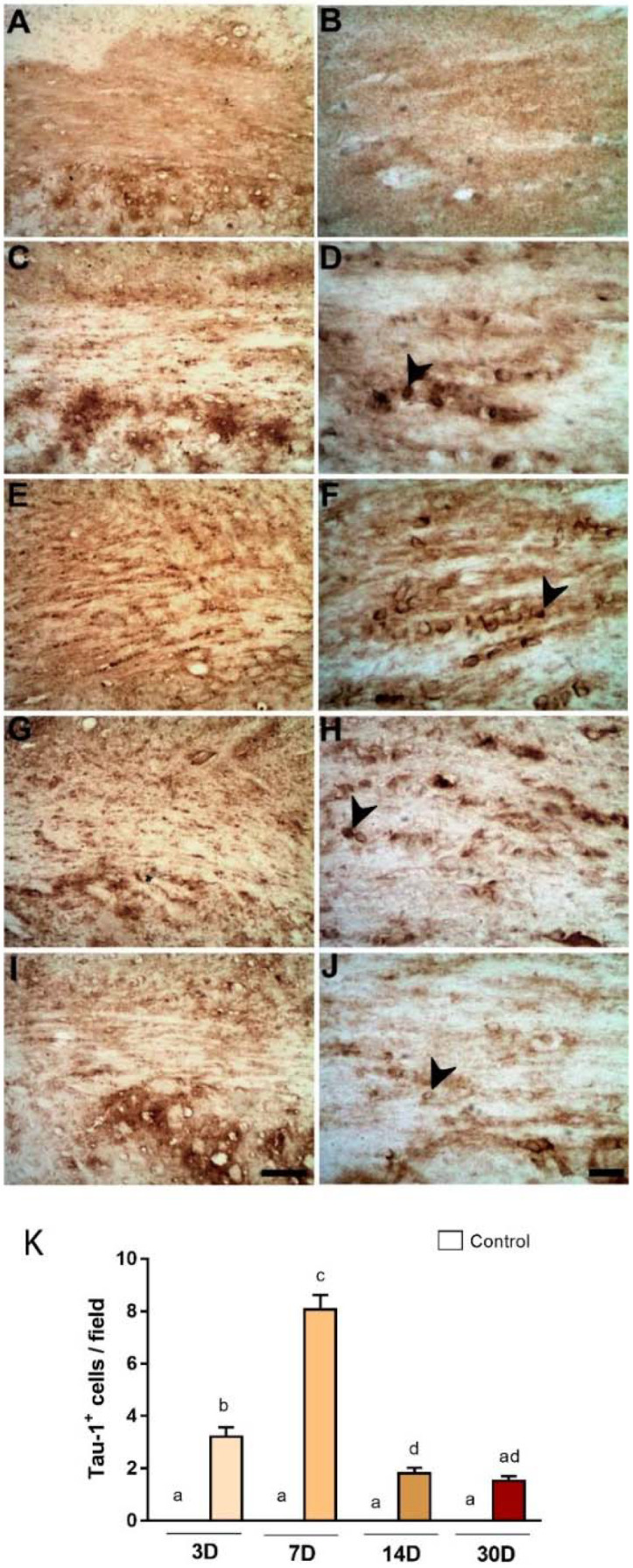
Increased pathological oligodendrocytes in the corpus callosum after striatal. Photomicrographs represent control animal injected with sterile saline (**A**,**B**) or ischemic animals injected with ET-1 at 3 (**C**,**D**); 7 (**E**,**F**); 14 (**G**,**H**); and 30 (**I**,**J**) PLDs. The results of quantitative analysis (**K**) are expressed as mean ± standard error of the mean (SEM). One-way ANOVA with Tukey’s post-hoc test (*p* < 0.05). Similar overwritten letters did not show significant statistical differences. Arrowheads point to TAU-1^+^ cells (pathological oligodendrocytes). Left-sided photomicrographs in lower magnification with a scale bar of 20 μm, and right-sided ones, with higher magnification with a scale bar of 100 μm.

TAU-1 is considered a reliable marker for pathological oligodendrocytes [27]. There was an increased expression of TAU-1 immunoreactivity in the CC of ischemic animals, with maximum immunoreactivity at PLD7 (Figure 4E,F). This has been confirmed by quantitative analysis (Figure 4K). Average numbers of TAU-1^+^ were 3.20 ± 0.36, 8.06 ± 0.55, 1.80 ± 0.22, and 1.53 ± 0.17 for 3, 7, 14, and 30 PLDs (Figure 4).

**Figure 5:**
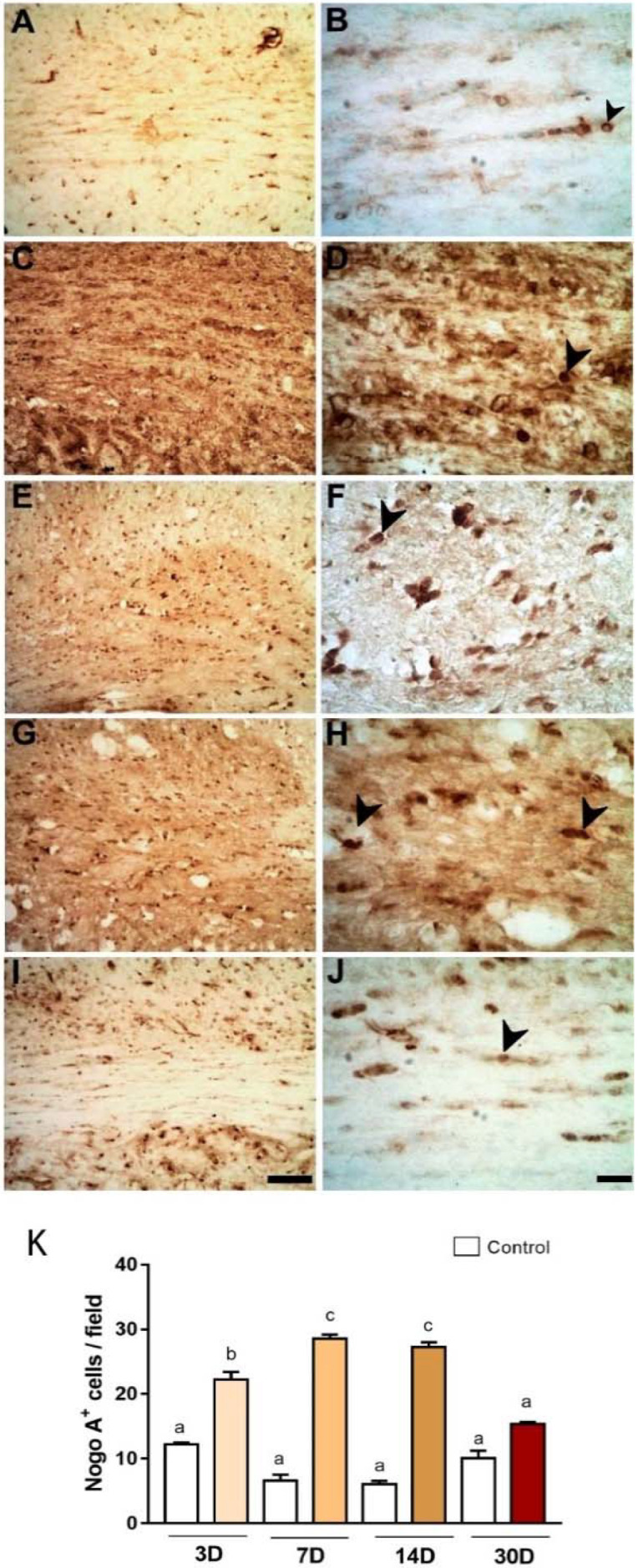
Nogo-A Immunoreactivity in the CC following striatal ischemia. Control animal injected with sterile saline (**A**,**B**) or ischemic animals injected with ET-1 at 3 (**C**,**D**); 7 (**E**,**F**); 14 (**G**,**H**); and 30 (**I**,**J**) PLDs. The results of quantitative analysis (**K**) are expressed as mean ± standard error of the mean (SEM). One-way ANOVA with Tukey’s post-hoc test (*p* < 0.05). Similar overwritten letters did not show significant statistical differences. Left-sided photomicrographs in lower magnification with a scale bar of 20 μm, and right-sided ones, with higher magnification with a scale bar of 100 μm.

We also investigated the expression of Nogo-A, a myelin-associated protein found to inhibit axonal regeneration [28], but also an oligodendrocyte marker [29]. In a qualitative inspection it was possible to notice an increase in Nogo-A immunoreactivity in ischemic animals in the time points evaluated (Figure 5: 3 (C-D) 3; 7 (E-F); 14 (G-H) 14; and 30 (I-J) 30 PLDs) when compared with control (Figure 5A,B). There was an increase in Nogo-A immunoreactivity in ischemic animals compared to control (*p* < 0.05, Figure 5K). In control animals, the average number of Nogo-A^+^ (cells/field) was 12.22 ± 0.25/field (Figure 5A,B). In ischemic animals, these numbers were 22 ± 1.18 (PLD3), 28.6 ± 0.60 (PLD7), 27.26 ± 0.75 (PLD14), and (15.53 ±0.79) (PDL 30). There were statistical differences between PDL7 and PDL-14 compared to control (Figure 5K, *p* < 0.05). PLD30 did not differ from control group (*p* > 0.05). Increased numbers between PDL3 and PDL7-14 were also statistically significant (*p* < 0.05). No statistical difference was observed between PLD7 and PLD14 (*p* > 0.05).

### 2.4. Microinjections of ET-1 into the Striatum Increase Astrocytosis in the Corpus Callosum

**Figure 6:**
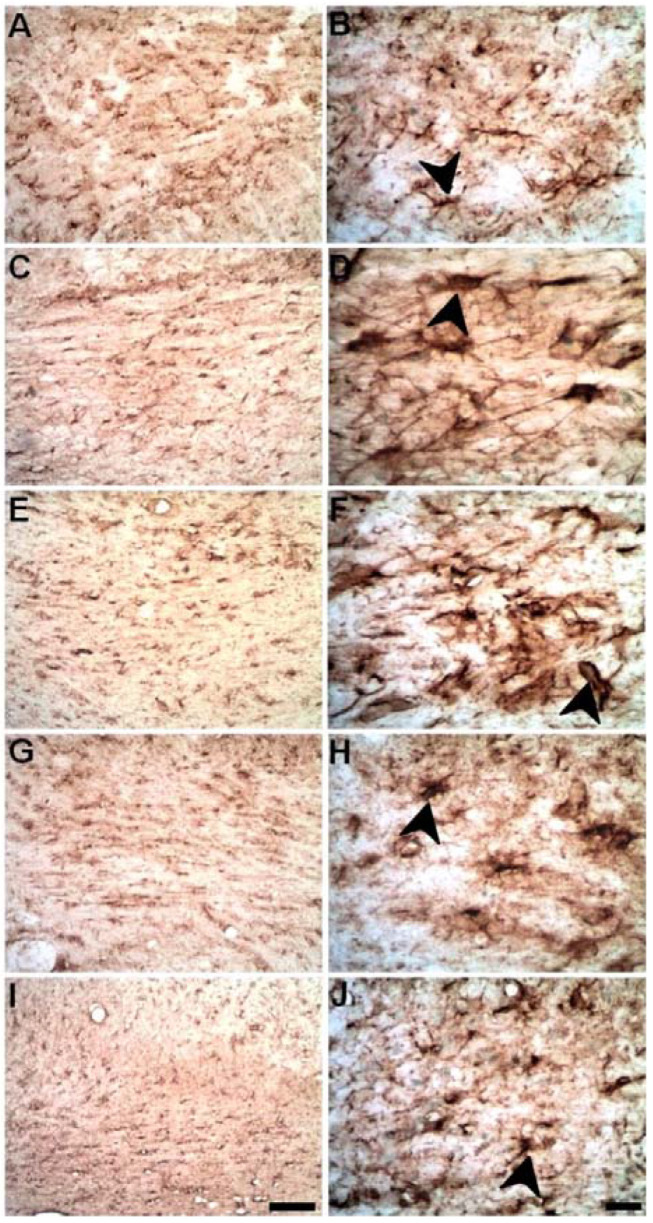
Progressive astrocyte activation as revealed anti-GFAP immunolabeling in the CC. Control animal injected with sterile saline (**A**,**B**) or ischemic animals injected with ET-1 at 3 (**C**,**D**); 7 (**E**,**F**); 14 (**G**,**H**); and 30 (**I**,**J**) PLDs. Scale bars: 20 μm (**A**,**C**,**E**,**G**,**J**) and 100 μm (**B**,**D**,**F**,**H**,**I**). Left-sided photomicrographs in lower magnification with a scale bar of 20 μm, and right-sided ones, with higher magnification with a scale bar of 100 μm.

Astrocytes were immunolabeled using anti-GFAP immunohistochemistry. There were no morphological changes in the astrocytes in the CC of control animals (Figure 6A,B), but progressive astrocytosis was observed in ischemic animals up to PLD14 with a decrease at PDL30 (Figure 6C–J).

### 2.5. Progressive Myelin Impairment in Rat’s the Corpus Callosum Secondary to Striatal Ischemia

**Figure 7:**
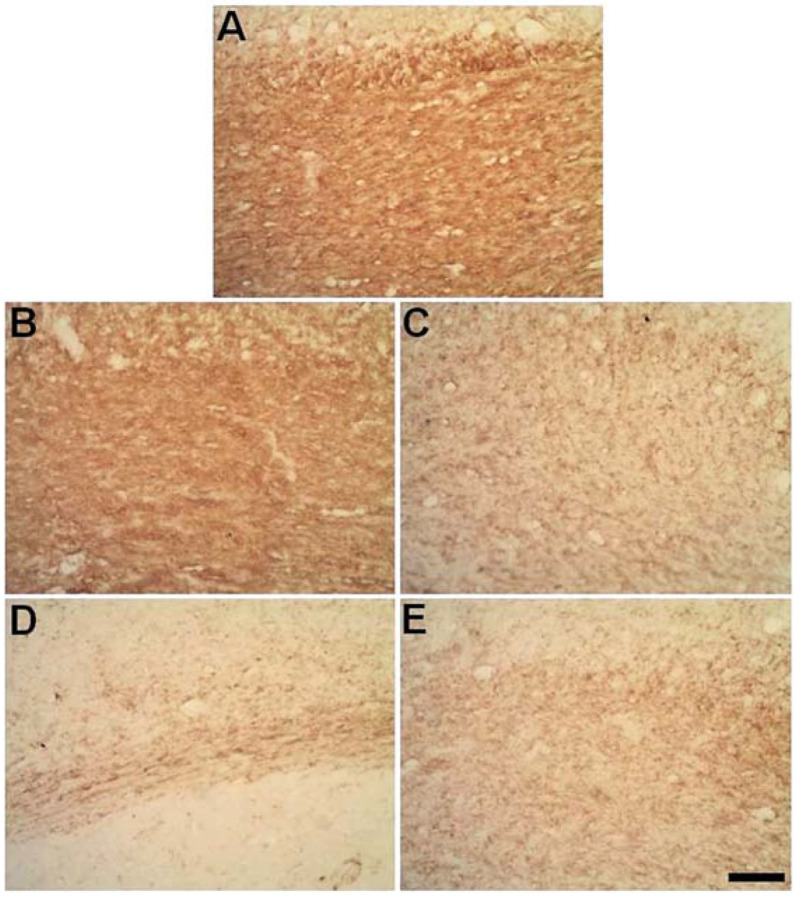
Myelin impairment in the CC secondary to focal striatal ischemia, as revealed by anti-MBP immunohistochemistry. Control animal injected with sterile saline (**A**) and ischemic animals injected with ET-1 at 3 (**B**); 7 (**C**); 14 (**D**); and 30 (**E**) PLDs. Decreased MBP immunoreactivity was present in later survival times. Scale bar: 100 μm.

Myelin sheath immunolabeling was performed using an anti-MBP antibody, a component of compact myelin [21]. There was a progressive decrease in MBP immunoreactivity in the CC of ischemic animals (Figure 7) compared to control. This myelin demise was more conspicuous between PLD7 and PLD30 (Figure 7C–E).

## 3. Discussion

In this study, we performed focal ischemic damage into the striatum in order to study secondary degeneration in the CC. There were conspicuous microgliosis and astrocytosis in the CC, followed by increased Nogo-A immunoreactivity, damage to oligodendrocytes, progressive myelin impairment, and later survival times. This parallels our previous studies using microinjections of NMDA into the rat SC ventral horn, in which secondary degeneration was present in the SC white matter tracts [20,21].

The ischemic damage was initially located in the striatum, not reaching CC. Nevertheless, in later time points, microgliosis and astrocytosis were present in the CC indicating secondary damage. This has important preclinical and clinical significance as CC is a WM commissure synchronizing functions between both brain hemispheres [26]. The importance of CC is inferred by the fact that callosal dysgenesis in humans is related to cognitive impairment, although neuroplasticity can minimize the deleterious effects [30].

In both trauma and stroke, primary damage is followed by a multitude of secondary events contributing to secondary degeneration and functional deficits [7,15,20,21]. As shown, primary damage was located in the rat striatum, but, in later survival times, CC damage was observed with conspicuous inflammation, oligodendrocyte, and myelin impairment. Microgliosis and astrocytosis are general pathological phenomena in response to CNS damage. Both phenomena possess beneficial and detrimental consequences in the pathological environment and neuropathological prognosis. Excessive microglial activation is detrimental and can contribute to secondary damage in both gray and white matter [12,31]. We have shown that microglial cells also expressed MHC-class II in the CC. This can be related to a neuroprotective phenotype [32]. In addition, it has been shown that expression of MHC class II in microglia is related to phagocytosis of WM tracts and not to a T cell response [33].

Minocycline treatment reduces microglial reactivity and is beneficial after middle cerebral artery occlusion in rats [34]. Similar results have been reported by our group after ischemic damage induced microinjections of ET-1 into the motor cortex [35] or striatum [36]. In future studies, minocycline treatment can be tested to avoid secondary degeneration of the CC as reported.

Astrocytosis was also present in different survival times following focal ischemic striatal damage and in the CC. We have previously shown differential patterns of astrocytosis in both grey and white matters following acute SCI in rats [20]. Astrocytosis is faster and more robust in the white matter than in the grey matter after following microinjections of NMDA into the ventral horn of the rat spinal cord [20]. Astrocyte reactivity can be both beneficial and detrimental following acute neural disorders, once in the late phase after stroke and trauma, reactive astrocytes form the glial scar, which isolates the lesion avoiding further damage, whichhis can also impair axonal regeneration [37,38,39]. Astrocyte also possesses different phenotypic profile with beneficial and detrimental actions [39]. It has been shown that microglia can release molecules that render a neurotoxic profile in astrocytes [39]. Further studies should clarify the role of astrocyte reactivity in the CC following focal striatal damage.

In this study, conspicuous WM degeneration was present in the CC mainly in later survival times. Pathological oligodendrocytes (Tau-1^+^ cells) were present in the CC from three days post-injury with peak at seven days. In a previous study [29], we have shown the presence of pathological oligodendrocytes in the ischemic striatum with a peak at three days. This later peak of pathological oligodendrocytes in the CC strongly suggests a second wave of oligodendrocyte death secondary to striatal damage. It is well established that pathological oligodendrocytes express the dephosphorylated Tau-1 following focal ischemia [27,29,40,41]. This is likely an attempt to preserve oligodendrocyte integrity in the early phase after ischemia [42], as Tau protein is a fundamental component of microtubule assembly [43].

As myelin impairment was present even 30 days post-injury, it is likely that damage of oligodendrocyte cell bodies precedes myelin demise, which is supported by our previous investigations using experimental models of striatal focal ischemia [29] and excitotoxic injury [16]. Progressive damage to oligodendrocytes has also been shown in the white matter tracts following SCI [20,44].

The data showed secondary neuroinflammation in the CC, which was coincident with degeneration of white matter tracts. We have previously shown that inhibition of microglia activation with minocycline preserves oligodendrocyte and myelin after acute striatal damage induced by NMDA-microinjections [16]. We hypothesize that an exacerbated microgliosis and astrocytosis might contribute to secondary degeneration of CC. This should be investigated in further studies using minocycline or another microglial inhibitor. This can be clinically relevant, as neuroinflammation in the white matter tracts is present even in longer survival times after striatal stroke in humans [17,45].

There was increased immunoreactivity for Nogo-A, a classic white matter inhibitor between 3- and 30-days post-injury with a peak at 7–14 days. In our previous study, immunoreactivity for Nogo-A in the ischemic striatum was higher at 3 days, decreasing thereafter [29]. In the present study, the maximum Nogo-A immunoreactivity in the CC was between 7 and 14 days indicating a later and increased expression of Nogo-A in the colossal tracts.

Other authors have shown that stroke induces increased immunoreactivity for Nogo-A in different survival times after CNS damage [46,47]. This increased immunoreactivity is likely related to the release of Nogo-A in degenerating myelin and oligodendrocytes. This should be related to a genetic program to reduce neuroplasticity in the pathological environment. Nogo-A is a potent inhibitor of axonal regeneration after both SCI [28,47] and stroke [46,47,48,49]. Inhibition of Nogo-A with specific monoclonal antibodies increases axonal regeneration after acute neural disorders [49,50,51]. Nevertheless, the function of Nogo-A after a stroke is much more complex than initially expected. It has been shown that expression of Nogo-A may contribute to the survival of neurons after ischemia [52]. Further studies should elucidate the meaning of increased Nogo-A immunoreactivity after a stroke.

Our results point out a progressive alteration in both glial cells and myelin in CC throughout the time points evaluated. Nevertheless, we must bear in mind that our findings should be interpreted in light of our model since models of hemorrhagic stroke or even ischemic models of artery occlusion can present distinct patterns of cell response [52]. Another important point to consider is related to the stroke location, which triggers distinct cellular responses [53]

Finally, neurological prognosis is highly influenced WM damage. It has been shown that excessive microglial activation and WM damage is a pivotal pathological finding after human stroke [17]. Ischemic stroke in the territory of middle cerebral artery (MCA) causes WM damage restricted to the striatum and some cortical regions. Nevertheless, it has been shown, using diffusion tensor imaging, progressive secondary microglial activation concomitant with anterograde degeneration of remote WM tracts up to six months following ischemic stroke in humans [17]. As long WM fibers convey both motor stimuli from the motor cortex to SC and sensory stimuli in the opposed direction, damage to these axons contributes significantly to neurological impairment and a poor clinical prognosis. In addition, in the reperfusion therapies for stroke, including thrombolysis and endovascular trombectomy, a recent meta-analysis suggested that WM, as named leukoaraiosis, is associated with poor prognosis in stroke patients in the 90 days outcome [54].

Degeneration of transcallosal fibers as found in the present paper may also influence motor outcome and neuroplastic events in the ischemic motor cortex in both experimental animals and humans [54]. This was clearly shown by Wang and colleagues. They used both diffusion and functional MRI to assess motor deficits and neuroplasticity in the cortical and subcortical motor areas. The results have shown callosal fibers connect higher-order sensorimotor areas and that degeneration of these fibers may impair motor performance and motor neuroplasticity [55].

## 4. Materials and Methods

### 4.1. Ethical Statement and Experimental Animals

Forty male adult Wistar rats (*Rattus novergicus*), weighing 250–290 g, were obtained from the Federal University of Pará (UFPA) animal facility. During the experimental period, animals were housed in a controlled temperature (25 ± 1 °C) room with a dark-light cycle of 12 h (lights on at 07:00 AM) in collective cages (with four animals each), with water and food ad libitum. This study was carried out in accordance with the Ethics Committee on Experimental Animal Research (CEUA) of the UFPA (protocol CEPAE-UFPA BIO-038-12), in agreement with NIH Guidelines for Use and Care of Experimental Animals. All efforts were made to avoid animal suffering and distress.

**Figure 8:**
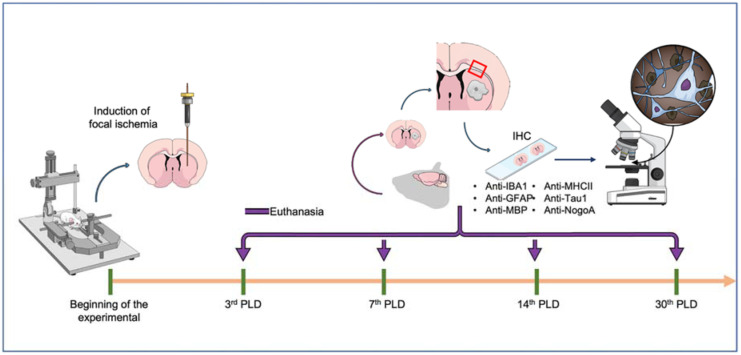
Schematic figure summarizing the experimental design. The focal ischemia was induced by endothelin-1 injection by a stereotaxic device with coordinated to striatum. Then, the animals were divided into four groups, considering different survival times after the ischemia (3, 7, 14, and 30 days), in which the brains were collected for immunohistochemistry analyses and further observations of the corpus callosum under bright-field microscopy.

The experimental animals were randomly distributed into five groups according to the experimental protocol. Figure 8 summarizes all methodological stages of the research.

### 4.2. Induction of Focal Striatal Ischemia

The stroke protocol was described in our previous study [29]. Briefly, animals (*n* = 5 per group) were initially anesthetized with a mixture of ketamine hydrochloride (72 mg/kg) and xylazine chloridrate (9 mg/kg) and, after the abolishment of their corneal reflex, were positioned in a stereotaxic frame (Insight, Brazil). Subsequently, small craniotomy was performed and 1 μL of ET-1 (80 pmol/μL; Sigma Company, St Louis, MO, USA) was injected into the dorsal striatum for 2 min, using a glass capillary micropipette (Sigma Company, St Louis, MO, USA) according to the following stereotaxic coordinates (in millimeters relative to bregma): 2.5 mm mediolateral; 1.2 mm anteroposterior; and 4.5 mm dorsoventral [56]. The pipette was then left stationary for 3 min before being slowly withdrawn. The control animals (*n* = 5 per paired group) were injected with 1 μL of vehicle (0.9% saline solution) following the same approach.

After surgical procedures, all animals returned to their cages. Four different survival times after lesion induction were considered in this experimental design: 3, 7, 14, and 30 PLDs. In the present stroke model, the mortality rate is less than 5%.

### 4.3. Perfusion and Histological Procedures

After appropriate survival times, all animals were anesthetized and perfused through the left ventricle with 0.9% heparinized-saline solution, followed by 4% paraformaldehyde in 0.2 M phosphate buffer (PB). After perfusion, brains were collected and post-fixed in 0.1 M PB for 24 h, followed by cryoprotection in increasing concentrations of sucrose-glycerol over 7 days. Cryoprotected brains were frozen in TissueTek^®^ (Sakura Finetek, Tokyo, Japan) and sectioned using a cryostat (Carl Zeiss/Micron, Jena, Germany). Coronal sections of 20 and 50 μm were mounted onto gelatinized slides and air-dried for 24 h. The slides were then stored in a freezer at −20 °C for posterior histological analyses.

#### 4.3.1. Immunolabeling Protocol

The immunohistochemical procedures were carried out following our previously published protocols [29]. In brief, the slide-mounted sections were removed from the freezer, kept in a heating oven at 37 °C for 30 min, and rinsed in 0.1 M phosphate buffer saline (PBS) for 5 min. After this, sections were pretreated in 0.2 M boric acid (pH 9.0) previously heated to 65 °C for 25 min for improving labeling intensity [20,21]. The slides were further allowed to cool down in borate solution for 20 min and were then incubated for the same time in 1% hydrogen peroxide in methanol under constant agitation. The slides were then rinsed three times (5 min each) in 0.05% PBS/Tween (Sigma Company, St Louis, MO, USA) and incubated with secondary antibody animal normal serum (see Table 1) in PBS for 1 h. Without additional rinsing, sections were then incubated with the primary antibody diluted in PBS for 24 h, rinsed three times in PBS/Tween solution for 5 min each, and incubated with the appropriate secondary antibody (Table 1) for 2 h. All incubations were made at room temperature (20 °C). As a negative control, PBS was used rather than the primary antibody in some randomly selected sections. Sections were rinsed three times again for 5 min and incubated in the avidin-biotin-peroxidase complex (ABC kit, Vector Laboratories Inc., Burlingame, CA, USA) for 2 h. Afterward, sections were rinsed four times (for 3 min each) and DAB-reacted. After DAB reaction, sections were rinsed three times (for 3 min each) in 0.1 M PB, dehydrated by alcohol, cleared in xylene, and coverslipped with Entellan^®^ (Merck, Darmstadt, Germany) [57].

#### 4.3.2. Qualitative and Quantitative Analyses

The ET-1 injection site was identified by the presence of tissue pallor associated with loss of cell bodies by basic histology revealed by Cresyl violet staining as described in our previous studies [29,37]. We surveyed under light microscopy (Nikon Eclipse E200, Tokyo, Japan), coronal sections containing corpus callosum above the primary area of ischemic lesion in the striatum. Adjacent 20 μm sections, anterior and posterior to the lesion site, were used for immunohistochemistry and all histological procedures. All stained sections were surveyed by light microscopy Illustrative images from all experimental groups were obtained using a digital camera attached to the microscope (Nikon Eclipse 50i, Tokyo, Japan) using the Moticam 2500 software (Motic Instruments Inc., Richmond, BC, Canada).

For evaluation of microglial activation, anti-Iba1 immunohistochemistry was used, as it recognizes a calcium-binding protein present in the cytoplasm of microglia [58]. This immunostaining allows recognizing morphological parameters of this glial cell: ramified (resting) and amoeboid microglial cell (activated). Myelin impairment was evaluated using an antibody against myelin basic protein (MBP), an important component of compact myelin [21,37].

For quantitative analysis, we counted the number of MHC-II^+^ cells, pathological oligodendrocytes (Tau-1^+^ cells), and Nogo-A^+^ cells per field. The counting field was defined as a square 0.25 mm wide grid (40x objective) in the eyepiece of the Nikon Eclipse 50i microscope above cited. All analyses were performed by two pathologists who were blinded to the treatments using the same optical microscope (Kappa coefficient > 0.7).

### 4.4. Statistical Analyses

The sample size was defined based on a previous study [37] and calculated by using free power analysis G^∗^Power software (version 3.1, Universität Düsseldorf, Dusseldorf, Germany) [59]. For this, the statistical power of 80%, an error of 5%, and predicting a sample loss of 5% were considered at the end of the study. After a descriptive statistic, comparisons among groups were performed using GraphPad Prism software (version 8.0, GraphPad Software Inc., La Jolla, CA, USA). The normality of results was tested by the Shapiro–Wilk method. The statistics difference among groups was analyzed with one-way ANOVA, followed by Tukey post-hoc test (*p* < 0.05). The results were expressed as mean ± standard error of the mean (SEM).

## 5. Conclusions

This study shows that secondary degeneration of CC occurs following focal striatal stroke. The secondary callosal damage is characterized by microgliosis, astrocytosis, oligodendrocyte damage, increased Nogo-A immunoreactivity, and progressive myelin impairment. These pathological events may impair callosal fiber functions, which might contribute to cognitive deficits. Future studies should use minocycline or another microglial inhibitor to modulate neuroinflammation in an attempt to preserve white matter. In addition, the use of specific antibodies against Nogo-A might be a desirable approach to increase callosal neuroplasticity.

## Figures and Tables

**Table 1:** Antibody details.

Primary Antibodies	Secondary Antibodies	Normal Serum (10%)	Labeling Purpose
Anti-MHC-II (1:100, Serotec, Oxford, UK)	Horse anti-mouse (1:100, Vector Labs, Burlingame, CA, USA)	Horse	Microglia
Anti-Iba-1 (1:1000, WAKO, Richmond, VA, USA)	Goat anti-rabbit (1:200, Vector Labs, Burlingame, CA, USA)	Goat	Microglia/macrophages
Anti-GFAP (GFAP, 1:1000, DAKO, Glostup, Denmark)	Goat anti-rabbit (1:200, Vector Labs, Burlingame, CA, USA)	Goat	Astrocytes
Anti-MBP (1:100, Serotec, Oxford, UK)	Horse anti-mouse (1:100, Vector Labs, Burlingame, CA, USA)	Horse	Myelin
Anti-Tau-1 (1:500, Chemicon, Temecula, CA, USA)	Horse anti-mouse (1:100, Vector Labs, Burlingame, CA, USA)	Horse	Pathological oligodendrocytes
Anti-Nogo-A (1:100, BD Transduction Lab, La Jolla, CA, USA)	Horse anti-mouse (1:100, Vector Labs, Burlingame, CA, USA)	Horse	Inhibition of axonal regeneration in oligodendrocytes

## Data Availability

All data are available within the article.

## Abbreviations

NMDA	N-Methyl-D-Aspartate
ET-1	Endothelin-1
CC	Corpus callosum
Cx	Cortex
St	Striatum
PDL	Post-Lesion Day
PBS	Phosphate buffer saline
SCI	Spinal cord injury
